# Transformational accounts of students’ undergraduate education are evoked by their engagement with knowledge

**DOI:** 10.1007/s10734-024-01332-9

**Published:** 2024-10-18

**Authors:** Paul Ashwin

**Affiliations:** Department of Educational Research, Lancaster University, Lancaster, UK

**Keywords:** Chemical engineering, Knowledge, Personal transformation, Student-as-consumer, Undergraduate education

## Abstract

There are strong concerns about students perceiving their undergraduate education in instrumental, rather than transformational, ways. However, it is not clear whether seeing education instrumentally undermines students’ capacity to see their education as transformational. Based on data from a 7-year longitudinal study of chemical engineering students from three countries, this article shows that all students focused on instrumental outcomes from education in their first year of study. However, by their final year, students tended to give instrumental accounts of what they had gained from their overall university experience and transformational accounts of what they had gained from studying their subject. This suggests that, depending on the context evoked, most students can describe instrumental or transformational relationships to their education. However, developing transformational accounts on their education appeared to be dependent on studying knowledge-rich degrees that supported them to engage with the world from the perspective of a particular body of knowledge. This raises serious questions about educational policies that imply that instrumental outcomes are the most important outcomes from students’ educational experiences as such policies obscure the importance of transformational knowledge-focused relationships that change the way that students engage with the world.

## The relationship between instrumental and transformational purposes of higher education

Debates around what the education offered by higher education is for have always focused on a variety of educational purposes (Ashwin, 2022). However, in a range of national and international policy contexts, the focus is increasingly solely on the development of employable graduates (Scott, 2022; Robson, 2023), particularly in Anglosphere nations (Lauder & Mayhew, 2020). In this way, higher education is positioned as an instrument that aims to develop student’s employability to achieve economic goals (education-as-instrument) rather than as an educationally focused institution that aims to transform students’ relationship with the world (education-as-transformation) (Ashwin, 2020; Biesta, 2022; Scott, 2022), even though perceptions of what ‘employability’ means for the education offered to students vary (Hooley et al., 2023).

The dominance of the education-as-instrument can be seen to be due to the strong influence of human capital theory on educational policy in Anglosphere contexts (Brown et al., 2020; Lauder & Mayhew, 2020; Wheelahan & Moodie, 2024). Human capital theory positions education as an investment, in which the human capital gained, usually understood as skills, leads to higher future earnings (Becker, 1964; Deming, 2022; in relation to higher education see Marginson, 2019). Such is the hold of human capital theory that when individuals’ investments in education fail to develop in the way predicted, policymakers have seen this as evidence of the failure of education, rather than the limitations of the theory (Brown et al., 2020; Moodie & Wheelahan, 2023).

The influence of human capital theory on higher education policy can be seen in England, where the quality and value of undergraduate degrees have been increasingly measured in terms of employment outcomes (Bradley & Quigley, 2023), and in the Australian ‘Job Ready Graduates’ policy, which attempted to direct prospective students into particular subject areas perceived to be connected to employment (Molla & Cuthbert, 2023). Internationally, the OECD (2017) and EU Commission have a strong focus on the labour market relevance of higher education. This focus can also be seen at the institutional level, where different universities increasingly promote a view of ‘graduateness’ based on broadly similar sets of generic graduate attributes. These are mainly focused on employability and do not take account of different forms of disciplinary or professional knowledge (for example, see Wong et al., 2022; Baron & McCormack, 2024). However, if higher education is about the development of generic skills, then it is not clear why undergraduate degrees should focus on supporting students to develop dynamic relationships with disciplinary, interdisciplinary and professional bodies of knowledge. The danger is that, given these dynamic relationships to knowledge tend to involve relatively expensive and inflexible traditional 3- or 4-year undergraduate degrees, the question is raised of whether generic skills might not be much more cheaply and accessibly delivered through the stacking of micro-credentials (Wheelahan & Moody 2022, Wheelahan and Moodie, 2024; Ljungqvist & Sonesson, 2023).

The limited view of education offered by human capital theory has been examined widely (Allais, 2012; Bernstein, 2000; Marginson, 2019; Wheelahan & Moodie, 2024). There has been less exploration of the way in which the human capital view of students’ relationship to knowledge is distorting. It presents the knowledge that graduates gain from their engagement in their undergraduate studies as a form of capital that is ‘banked’ (Brown et al., 2020), which positions students outside of this knowledge and obscures the ways in which students’ relationships to knowledge differ between subjects (Ashwin, 2020). This view underpins the education-as-instrument view of education, in which education is the instrument for the production of capital. This is in contrast to the education -as-transformation view that foregrounds how higher education supports students to see the world from inside particular bodies of knowledge, which changes students and the ways in which they engage with the world (Taylor, 1993; Bowden & Marton, 1998; Ashworth, 2004; Dall’Alba & Barnacle 2005; Ashwin, 2020).

In positioning students outside of knowledge, the education-as-instrument view positions students as instrumental consumers who are primarily focused on gaining credentials rather than engaging personally with knowledge (for example, see Naidoo & Jamieson, 2005; Molesworth et al., 2009; Neary & Winn, 2009; Saunders, 2015; Brooks, 2018; Gunn, 2023). This informs a marketized vision of higher education found in policy documents in which a high-quality undergraduate education system is positioned as one shaped by student choice, that provides students with employability skills and produces employable graduates (Ashwin et al., 2015). It positions the value of a degree simply in terms of what it allows students to do after graduation, its exchange value (McArthur, 2011; Tomlinson 2018, Tomlinson & Watermeyer, 2022), rather than valuing how students have been personally transformed through their engagement with knowledge (Ashwin, 2020).

A critical question is whether students’ instrumental relationships to their education can reduce the likelihood that they will engage with their education in transformational ways. Part of the fear of those who decry student consumerism is that it will eventually erode students’ intellectual commitment to knowledge. For example, Bernstein (2000, pp. 69–70) identifies the dangers of a ‘De-Centred Market Pedagogic Identity’ in which the focus is on meeting the short-term needs of the labour market and personal commitments to knowledge are seen as a barrier to the free flow of knowledge. The concern is that students’ consumer attitude will undermine their academic engagement. There is evidence that an instrumental focus and a lack of personal engagement with knowledge have a negative impact on the quality of students’ engagement with their education (Finney & Finney, 2010; Tomlinson 2017; Brooks & Abrahams 2018; Nixon et al., 2018) leading to lower levels of academic performance (Bunce et al. 2017; Bunce & Bennett, 2021). But it is not clear whether the lack of personal engagement is due to an instrumental focus limiting the transformational potential of students’ educational experiences. In order to understand whether education-as-instrument can undermine education-as-transformation, it is necessary to understand how they are related within students’ educational experiences. There are four ways in which this could occur.

First, different students could have different orientations to higher education with some being focused on education-as-an-instrument to gain credentials for employment and some focused on education-as-transformation (for example Brint, 2012; Spronken-Smith et al., 2015; Willner et al., 2023; Schafer 2024). However, there is strong evidence that this distinction is too crude, and the same students can see their education as both instrumentally valuable and personally transformational (for example Tight 2013; Budd 2017; Patfield et al. 2021; Reynolds 2022; Ashwin et al., 2023; Gupta et al., 2023; Mendes & Hammett, 2023; Taylor Bunce et al., 2023).

Second, rather than differences between students, the idea of ‘prosumption’ suggests that students’ roles may be made up of different combinations of instrumental and transformational elements (Dusi & Huisman, 2021). Third, rather than students’ roles having a mixture of instrumental and transformational aspects, students could have either instrumental or transformational relationships with different aspects of their education (Ashwin et al., 2023). For example, they may have an instrumental relationship with their institution but have a transformational relationship to the knowledge they are studying. A fourth explanation is that education-as-instrument and education-as-transformation offer different kinds of description of students’ educational experiences rather than being related to each other. This explanation would suggest that, for students, an instrumental or transformational description of their educational experience can be ‘evoked’ by their understanding of their context in a similar way to the manner in which different conceptions of learning can be evoked depending on students’ understanding of their educational context (Trigwell & Ashwin 2006; Ashwin & Trigwell, 2012; Trigwell et al., 2013). Under this understanding, some situations would call for students to give an instrumental account of their educational experiences and others a transformational one, without the different accounts necessarily impacting on each other.

In this article, I explore the relationships between education-as-instrument and education-as-transformation in students’ accounts of their education during the 4 years of their undergraduate degrees. I draw on a 7-year longitudinal study which focuses on students who studied chemical engineering in two English, two South African and two USA universities. There are four elements of this study that make it a revealing case to examine the relationships between students’ accounts of education-as-instrument and education-as-transformation. First, chemical engineering is one of the subject areas with the highest salaries for graduates (Quadlin et al., 2023) and students often cite the career prospects offered by Chemical Engineering as a key reason for deciding to study it (Godwin et al., 2014; Mathebula, 2018). Second, in relation to knowledge, engineers, as well as scientists, are more likely than students from other subjects to be fact orientated (Lonka et al., 2021) and students studying STEM subjects are more likely to adopt consumerist perspectives on their education than students from other subjects (Bunce et al., 2017). Third, England, South Africa and the USA are argued to be paradigmatic cases of marketized HE systems (Czerniewicz et al., 2023; Durán Del Fierro, 2023) and reflect the instrumental view of education that is argued to have become embedded in the Anglosphere (Lauder & Mayhew, 2020). All involve students paying tuition fees which are a key element in the development of student consumerism (Plamper et al., 2023), although it should be recognised that higher education in South Africa, in response to its apartheid history, is much more explicitly positioned in policy as having a role in transforming society than in England or the USA (Boughey & McKenna, 2021). This has been found to relate to the reasons that some students study engineering in South Africa (Jawitz & Case, 1998). These four factors suggest that students studying chemical engineering in England, South Africa and the USA are in a situation in which the elements supporting education-as-instrument perspective might be at their highest.

The longitudinal design of this study is also important. Most studies of how students and graduates understand their relationship with their higher education to draw on one moment of data collection (for example Case et al. 2018; Muddiman 2020; Ashby-King & Anderson, 2022; Brooks et al., 2022) rather than offering an insight into the extent to which these are stable over time.

## Methods

### Research design

This article draws on data from a larger project examining students studying undergraduate degrees of chemistry and chemical engineering in two universities in England, two in South Africa and two in the USA. We tracked students for 7 years in total from their first undergraduate year of study to up to 4 years after graduation depending on the length of their degree and the time it took them to complete it. The methodology of this project owes a considerable debt to a previous project examining sociology (see McLean et al., 2018). This article reports on data from the longitudinal study of chemical engineering students tracked through their undergraduate degrees in the three countries.

### Data generation and analysis

All institutions and participants were anonymised in line with the ethical approval granted by the lead institution in the research (Reference Number FL15035). Ethical approval was also obtained as required at each of the research sites. The universities in this research were given pseudonyms based on chemical elements. These were:

- England — Erbium University and Europium University
- South Africa — Samarium University and Sodium University
- USA — Argon University and Astatine University

The data for this article were drawn from interviews with students studying chemical engineering. An earlier article (Agrawal et al., 2024) analysed the differences in the curricula in terms of differences in contact hours, curriculum rigidity and structure and showed that the programmes involved an intense engagement with disciplinary knowledge. In the first year of their degrees, we initially interviewed a self-selecting sample of 102 students (34 England, 38 South Africa, 30 USA). We then sought to identify 60 (10 from each institution) students from each degree programme that reflected its diversity in terms of ethnicity and gender. We interviewed students in each year of their undergraduate degree. We have only included students in the analysis for this article if they had completed an interview in the first or second year and final year of their undergraduate degrees. This reduced the sample from 60 to 48 students who had sufficient data to be included in the analysis for this article, 20 from England, 19 from South Africa and 9 from the USA. Due to differences in the minimum length of the degree (3 years in England and 4 years in South Africa and the USA) and differences in time students took to complete their degrees, the final undergraduate year was the third year interview for 16 students (all from the English institutions), the fourth interview year for 27 students, the fifth year interview for four students, and the sixth year interview for one student. In total, we had 165 interviews with these students during their undergraduate studies, with the number of interviews with each student ranging from 2 to 6. Students were given pseudonyms reflecting the cultural diversity of the cohorts.

The semi-structured interviews with the students normally lasted between 60 and 90 min. They followed a common protocol with questions covering students’ background, route into university, study practices, understanding of disciplinary knowledge. In the interviews, students were asked about why they had chosen to study chemical engineering, what they saw themselves doing in 5 years’ time and what they wanted to gain from studying chemical engineering and from going to university. Although the analysis drew on the full interview transcripts, it was in response to these questions that students particularly focused on outlining what they wanted to get out of their degrees.

It is important to note that the question about what students gained from studying chemical engineering was part of a section of the interview in which we asked them about their views of knowledge about chemical engineering and what it meant to them to be a chemical engineer. The question about what students gained from going to university was in a later section of the interview where they were asked about their attitude towards their university and the value of a university degree.

### Data analysis

Initially, as with similar analyses (Ashwin et al., 2016, 2023), a phenomenographic approach (Marton & Booth, 1997) was taken to analysing the data. Phenomenography is a way of analysing data that seeks to capture the variation in the way that a group of people experience a phenomenon. Rather than applying theory to the data or using a priori categories to structure the analysis, a phenomenographic approach seeks to establish all the different ways of seeing that phenomenon that are expressed in the data and to place them in a logical and inclusive hierarchical structure (Åkerlind, 2005; Marton & Booth, 1997).

The analysis focused on the qualitative variation in students’ accounts of what they wanted to gain from studying chemical engineering at university. This involved identifying the most important things that students were seeking to gain from studying across all of their interviews. Once these were identified, the intention was to form an inclusive hierarchy of what students wanted to gain. However, once all of the elements had been identified, it became clear that they did not form the inclusive hierarchies that are usually generated through phenomenographic analysis (Åkerlind, 2005; Marton & Booth, 1997). This appeared to be because when students discussed what they wanted to gain from studying they were focused on different phenomena rather than having different perceptions of the same phenomenon. For example, some participants focused on their qualification, some focused on their educational experiences, and others focused on what they could do after they graduated. Thus, whilst the data were analysed phenomenographically and produced categories of description, these categories of description did not form an inclusive hierarchical outcome space expressing the relations between the categories.

In reporting the outcomes, an insight into both the structure of the outcomes is given by the use of simple numerical counts, and the meaning of the outcomes, by the use of quotations from individual students.

## Outcomes

### What students hoped to gain by going to university to study chemical engineering in their first year

In the interviews in students’ first year of study, the reasons that students gave for going to university and for studying chemical engineering were consistent. They answered both questions as if they were a single question about going to university to study chemical engineering. They outlined three such reasons: to develop a career, to become a chemical engineer, and to be able to make a contribution to society as a chemical engineer. There were roughly equal numbers of students with gave each of these reasons.

Sixteen of the 48 participants were studying chemical engineering to support the development of a career, which was not explicitly related to chemical engineering. For example, Anika focused on how studying chemical engineering would give her the option of going into medicine or the cosmetics industry:

I figured with a chemical engineering degree I have two options that I really enjoy, where I could go into medicine later in the future, which I have still not decided yet. Either go into medicine, or go in into the cosmetic industry, which is another big passion of mine. (Anika, Argon, Year 1).

Fourteen of the 48 participants said they were studying chemical engineering specifically to become a chemical engineer. Unlike the students in the previous category, they did not mention pursuing a career outside of chemical engineering and they were clear that they were studying chemical engineering as a necessary step in becoming a chemical engineer:

Because for chemical engineering, that’s the necessary path, there’s not a trade school for chemical engineering more of. So, I feel like that’s the only route... There’s no other way you could be a chemical engineer without a university degree. So that’s it, one option. (Janja, Astatine, Year 1).

Eighteen of the 48 participants were studying chemical engineering so that they could contribute to improving society as a chemical engineer. This reason was more common amongst the students in South Africa with 13 of the 18 from South Africa.

To be able to go out, hopefully find a job, hopefully in a job that involves something to do with helping the environment, improving the lives of some people. Maybe desalination if that ever happens here because of the drought, if the drought continues. Or wastewater treatment or maybe good pharmaceuticals, mass producing vaccines, something like that. Who knows? I prefer to work where it would help people or help the environment rather than where it would generate more money if that were a thing (Naas, Samarium, Year 1)

All three of these initial reasons are based on an education-as-instrument view of what students expect to gain with their education. There is variation in what this instrument being used; individual benefit, in the case of developing a career and societal benefit in the case of contributing to society as a chemical engineer. However, in each case, education is positioned as an instrument to achieve these outcomes.

### What students gained from going to university to study chemical engineering in their final year

In their final year of studying, what students identified a gaining from going to university was different from what they identified as gaining from studying chemical engineering. Most students focused on instrumental gains from going to university and transformative outcomes from studying chemical engineering.

In relation to what they gained from going to university, students focused on three outcomes: a degree; the opportunity to continue their education; and an opportunity to contribute.

Nearly all of the participants (43 out of 48) identified the degree that they would graduate with as the most important thing that they had gained because of what it would allow them to do in the future:

That’s a good question. To start off with, a degree, which is obviously a big qualification to have. Even if I don’t go into chemical engineering, it’s still like something to say this person has achieved something (Leon, Erbium, Year 4)

These participants tended to express appreciation for the experiences and friends they had at university but were clear that the degree was the first thing they would emphasise:

At the end of the day, obviously a degree number one. I think, to walk away with relationships and friends that I know I’m going to have for the rest of my life. Thankfully, I can confidently say that I will walk away with that. To be able to walk away as a friend of people, build relationships, and also have the technical background that I need to go into industry and to be successful in industry (Alexander, Argon, Year 5).

Four of the 48 participants focused on the transformational educational experience of studying for a degree as the most important thing they had gained from university:

Just an experience that will help me in the future and will teach me a lot. Uni changes people, and I completely understand that now…I think it makes you grow, and it really challenges you. It’s not just your capability in a subject, or anything like that. I think it’s just basic life skills, like time management, independence, dependence. (Rubiya, Europium, Year 4)

One student focused on the contribution that they would be able to make after graduating as the most important thing that they had gained from going to university:

I hope to be able to help solve problems that are already existing. And I can reach out to and can be able to solve. Because I feel like our tasks as engineers are mostly problem solving. And that is what I want to go into, be it with skincare disorders or nutritional benefits from getting organic food. I want to solve an already existing problem (Nomathemba, Samarium, Year 4)

In relation to what they gained from going to university, nearly all the students focused on the instrumental value of their degree rather than the transformational aspects of their education. This was in contrast to what they gained from studying chemical engineering where students tended to focus on how their engagement with knowledge had changed the way they saw and interacted with the world. Students talked about three things they gained from studying chemical engineering: a way of engaging with the world; an understanding of chemical engineering; and access to a career.

Over three-quarters (37 out of 48) of the participants focused on the way of engaging with the world that they had gained from studying chemical engineering. There were three different ways in which students expressed this new way of engaging with the world but, in each case, there was a sense that they were seeing this knowledge from the inside and using it to inform their engagement with the world. For eight participants, it was a new way of engaging with the world with rigour and perseverance that was the most important thing they had gained from studying chemical engineering:

Interestingly enough, I would not say the technical knowledge. I would say the perseverance and the, the self-discipline to just work hard. I feel that I can apply it to anything. I can apply it to music and mastering a new instrument if I want. I can apply it to relationships to know that things aren’t going to go well all the time and yes, you need to be able to evaluate, in the long term, is this worth what it is, or... is it what you want? (Thomas, Sodium, Year 4)

For 24 of the participants, it was thinking as an engineer that was key:

It’s the thought process, the mind-set. I think, basically, engineering judgement. I don’t know if it’s the most important skill but it’s the skill I value the most, I think it’s the most valuable. (Nabeel, Samarium, Year 5).

For five participants, four of whom were studying in South Africa; it was about a new way of engaging with the world that help to contribute to the addressing of societal issues:

Appreciation. I’m starting to really appreciate a way of life as a society. It then gives me a very, very good platform to actually help out in society, a very good platform. I’m especially interested in energy and energy production because energy, essentially, is what fuels our everyday lives. That’s why chemical engineering is very important to me. The fact that, as I said, it’s the direct link between our home and our everyday lifestyle and protecting our home obviously is very important or else we can’t enjoy our everyday lifestyle. (Lekan, Erbium, Year 3)

Eight participants focused on the understanding they had developed of chemical engineering. However, in contrast to the previous category, they spoke about this knowledge as if they were engaging with it from the outside. There was no sense that it had transformed their ways of engaging with the world:

Understanding, I think, as in understanding the actual discipline of chemical engineering. There are so many things I’ve learnt, but I can’t even think of them. Independence, definitely, and being confident in your own work. That is, I think, 100%. (Rubiya, Europium, Year 4)

Three participants focused instrumentally on how studying chemical engineering had helped them to be successful in their careers:

I feel like it’s definitely helped me to get a job in the industry in terms of my knowledge so if I apply this knowledge to industry, you can see how you could use it. It’s been interesting as well as that because obviously when you pick the degree, chemical engineering, you’re thinking in your head, “Well I’m probably going to go into the chemical engineering industry at the end of it,” because that’s what you’re learning. (Lincoln, Erbium, Year 4).

When discussing what they had gained from studying chemical engineering, a substantial majority of students focused on how they had gained a new way of engaging with the world by viewing it from the perspective of chemical engineering. Some participants focused on the knowledge they had gained from chemical engineering but still gave the sense they were seeing this knowledge from the outside and it had not changed their way of engaging with the world. A small number of students focused on how their degree had helped them be successful in their careers and appeared to be focused on the instrumental value of their degree rather than the benefits of their personal commitment to knowledge.

### Relations between students’ initial reasons for studying, what they gained from being a university and what they gained from studying chemical engineering

Table 1 shows a consistency between the education-as-instrument views of students in their first year and what they had gained from going to university in their final year. However, Table 2 shows that, despite all students being focused on gains related to education-as-instrument in their first year, in their final year most students focused on gains relating to education-as-transformation in relation to their studying of chemical engineering.

**Table 1:** What students hoped to gain in their first year compared to what they gained from going to university in their final year of study

	What students gained from going to university in their final year			
What students hoped to gain in their first year	*A degree (I)*	*Process (T)*	*Contribute (T)*	Total
*Establish a Career (I)*	14	2	0	16
*Become Chemical Engineer (I)*	13	1	0	14
*Contribute to Society as Chemical Engineer (I)*	16	1	1	18
Total	43	4	1	48

(I) refers to education-as-instrument gains in which the account focuses on how education will be used as an instrument to achieve other outcomes

(T) refers to education-as-transformation gains in which the account focuses on how education has transformed the students’ perspective

**Table 2:** What students hoped to gain in their first year compared to what they gained from studying chemical engineering in their final year of study

	What gained from studying chemical engineering in their final year			
What students hoped to gain in their first year	*Career (I)*	*Knowledge of chemical engineering (I)*	*Way of engaging (T)*	Total
*Establish a Career (I)*	0	4	12	16
*Become Chemical Engineer (I)*	2	2	10	14
*Contribute as Chemical Engineer (I)*	1	2	15	18
Total	3	8	37	48

(I) refers to education-as-instrument gains in which the account focuses on how education will be used as an instrument to achieve other outcomes

(T) refers to education-as-transformation gains in which the account focuses on how education has transformed the students’ perspective

Table 3 shows that most students who focused on education-as-instrument when discussing what they gained from going to university in their final year, focused on education-as-transformation when discussing what they had gained from studying chemical engineering.

**Table 3:** What students gained from studying chemical engineering compared to what they gained from going to university in their final year of study

	What gained from going to university			
What gained from studying chemical engineering	*A degree (I)*	*Process (T)*	*Contribute (T)*	Total
*Career (I)*	3	0	0	3
*Knowledge of chemical engineering (I)*	6	2	0	8
*Way of engaging (T)*	34	2	1	37
Total	43	4	1	48

(I) refers to education-as-instrument gains in which the account focuses on how education will be used as an instrument to achieve other outcomes

(T) refers to education-as-transformation gains in which the account focuses on how education has transformed the students’ perspective

For example, Liam was clear that he gained a way of thinking from studying chemical engineering and a degree for use in his career from going to university.

Interviewer: What are the most important things that you’ve gained from studying chemical engineering?

Liam: I think definitely the way of thinking … just in terms of the broadness of it, to learn about lots of different things, the way of thinking and the way to deal with working as an engineer kind of thing has, kind of, set you up quite well to then move on into the real-world, which will probably be completely different.

...

Interviewer: What is it that you hope to gain from being at Erbium?

Respondent: I think a good reputable degree, I suppose, that can then be used in my future career to getting jobs and things like that, and then, ultimately, progress through chemical engineering

(Liam, Erbium Year 4).

A small number of students focused on the instrumental elements of both what they gained from studying chemical engineering and what they gained from going to university. For example, Leo saw both in terms of his career:

Interviewer: What are the most important things that you feel you’ve gained from studying chemical engineering?

Leo: Hopefully it gets me a career (laughter). Yes, a career that will hopefully give me plenty of variation in what I do and I can be quite comfortable living, I think that’s mostly what I’m getting out of it.

...

Interviewer: Okay. What is it that you actually hope to gain from being at Erbium?

Leo: I hope it’s given me the skills to exist in adult life and set me up to be employable in the future in the field of chemical engineering.

(Leo, Erbium Year 4).

There were no clear institutional differences in students’ accounts of their initial reasons for studying, what they gained from going to university or what they gained from studying chemical engineering. The only clear national difference was, as noted earlier, that nearly all the students who initially studied chemical engineering to contribute as chemical engineer, and all the students who felt they had developed a way of engaging with the world that would allow them to contribute to society, were studying in South Africa.

## Discussion

What are we to make of the apparently contradictory findings of this study? Table 1 appears to tell a depressing story of students being instrumentally focused on simply gaining a degree throughout their time at university. However, Table 2 suggests that most students reported gaining a new way of engaging with the world from studying chemical engineering despite their initial instrumental reasons for studying chemical engineering. This tells a much more optimistic story of students being transformed by knowledge through their studies. Table 3 indicates that being instrumentally focused on gaining a degree from going to university went alongside the transformational value of gaining a new way of engaging with the world from studying chemical engineering. There are three aspects of these outcomes to highlight in making sense of them.

First, in contrast to some previous research (for example Brint, 2012; Spronken-Smith et al., 2015; Willner et al., 2023; Schafer 2024), these findings suggest that it is not the case that some students are instrumentally focused whereas others are focused on transformation. It also appears that students’ accounts of what they gain from engaging with higher education are not related to their initial reasons for studying.

Second, it suggests that when reflecting on what they gained from going to university, most students gave a response consistent with education-as-instrument that focused on the exchange value of a credential rather than their personal engagement with knowledge (Naidoo & Jamieson 2005; Molesworth et al., 2009; Neary & Winn, 2009; Saunders, 2015; Brooks 2018). However, when reflecting on what they gained from studying chemical engineering, students focused on how the bodies of knowledge they had studied transformed their ways of engaging with the world. This suggests that, rather than consumerist discourses necessarily undermining students’ commitment to knowledge (Finney & Finney 2010; Bunce et al. 2017; Tomlinson 2017; Brooks & Abrahams, 2018; Nixon et al., 2018; Bunce & Bennett, 2021), students’ reflections on what they gained from higher education was shaped by the context that was evoked when they are constructing their account (Trigwell & Ashwin 2006; Ashwin & Trigwell, 2012; Trigwell et al., 2013). This suggests that rather than students’ roles being made up of instrumental and transformational elements (Dusi & Huisman, 2021), they are better understood as different kinds of accounts that students can give about their education depending on the context evoked when they give their account.

Third, it is important to note, that in their first year, all students saw questions about what they wanted to gain from studying chemical engineering and studying at university as the same question, which called for an education-as-instrument informed answer. By their final year, most students saw questions about studying chemical engineering as requiring an education-as-transformation informed answer, whilst still seeing what they gained from university in instrumental terms. This suggests that it was the knowledge-rich context of studying for their chemical engineering degrees that evoked transformative student accounts of their educational experiences (Taylor, 1993; Bowden & Marton, 1998; Ashworth, 2004; Dall’Alba & Barnacle 2005; Ashwin, 2020). Therefore, such accounts may not have been evoked if they had studied a degree that did not support them to use such knowledge to engage with the world. It appears it is the *absence* of knowledge-focused education that undermines education-as-transformation rather than the *presence* of education-as-instrument accounts.

It should be noted that around a fifth of students offered only instrumental accounts when talking about what they had gained from their degree and what they had gained from studying chemical engineering. However, this is lower than might be suggested by those concerned about student consumerism (Molesworth et al., 2009; Nixon et al., 2018) for a degree known for the highest graduate salaries (Quadlin et al., 2023) in highly marketized higher education systems (Czerniewicz et al., 2023; Durán Del Fierro, 2023).

## Conclusion

These outcomes have six important implications for future research, educational practices and educational policies.

First, these findings underline the dangers of the way human capital theory has underpinned the logic of policies focused solely on the development of employable graduates highlighted by some researchers (Brown et al., 2020; Scott 2022; Moodie & Wheelahan, 2023; Robson, 2023; Wheelahan & Moodie, 2024). These findings show the importance of knowledge-rich education in supporting education-as-transformation and highlight the danger of policy contexts that promote the value of 'graduateness' simply in terms of the employability of graduates without taking seriously the forms of knowledge that produce graduateness (Ashwin, 2020; Bradley & Quigley, 2023; Molla & Cuthbert, 2023; Robson, 2023).

Second, these findings highlight that knowledge-rich, education-as-transformation versions of graduateness are important because they emphasise how graduates see and engage with the world from inside particular bodies of knowledge (Taylor, 1993; Bowden & Marton, 1998; Ashworth, 2004; Dall’Alba & Barnacle 2005; Ashwin, 2020) whereas education-as-instrument versions simply focus on what students as individuals can expect to gain from higher education. Views of graduateness that ignore the knowledge that students have engaged with are essentially empty (Bernstein 2000; Ashwin et al., 2015; Hooley et al., 2023), which reinforces the serious questions that are being asked about whether a knowledge-rich education can be offered through the stacking of micro-credentials (Wheelahan & Moody 2022, 2024; Ljungqvist & Sonesson, 2023).

Third, these findings suggest that universities are mistaken in presenting the outcomes of their degrees in terms of generic graduate outcomes (Wong et al. 2022; Baron & McCormack, 2024). Universities should emphasise their role as educational institutions by developing and foregrounding education-as-transformation accounts of how students benefit from engaging with knowledge rather presenting an education-as-instrument view of degrees as mechanisms for achieving economic goals (Ashwin, 2020; Biesta, 2022; Scott, 2022).

Fourth, the way in which it was overwhelmingly the South African students who focused on contributing to society as a chemical engineer, highlights the importance of societal conversations about the purposes of higher education. A similar focus was found in an earlier study of South African students studying engineering (Jawitz & Case, 1998) and it suggests that these student accounts reflect the role that South African higher education is expected to have in transforming society (Boughey & McKenna, 2021).

Fifth, the study highlights that great care has to be taken when using student accounts to make claims about the impact of student consumerism on students’ educational experiences. As such accounts change according to the context evoked, studies need to be aware of the context that has been evoked for students when asking them to reflect on their educational experiences. Whilst this is a challenge for all such studies (for example Finney & Finney 2010; Bunce et al. 2017; Brooks & Abrahams, 2018; Nixon et al. 2018; Bunce & Bennett, 2021), it would appear to be even more challenging for survey-based studies as it is harder to discern the context evoked when students respond to closed survey items.

Finally, whilst there is danger in policy discourses promoting a very limited and limiting idea of graduateness, these outcomes also suggest that such discourses can never be totalising. Even where most students understood what they had gained from going to university in instrumental terms, there were still a small number of students who foregrounded transformational aspects of going to university. As Bernstein (2000) emphasises, these discourses do not completely structure the possibilities for students. However, this should not offer cause for complacency. The outcomes of this study emphasise the importance of students, universities, policymakers and wider societies understanding that the benefits of undergraduate education are dependent on students’ engagement with disciplinary bodies of knowledge.

## Data Availability

The data reported in this article will be deposited with the UK Data Service once the research is complete.

## References

[CR1] Agrawal, A., Carroll, J., Blackie, M., Rosewell, K., & Pitterson, N. (2024). Impact of curricula on student learning: A comparison of six chemical engineering programmes in three Washington Accord countries. *Southern Journal of Engineering Education*, *3*(1). 10.15641/sjee.v3i1.1474

[CR2] Åkerlind, G. (2005). Variation and commonality in phenomenographic research methods. *Higher Education Research & Development,**24*, 321–334.

[CR3] Allais, S. (2012). ‘Economics imperialism’, education policy and educational theory. *Journal of Education Policy,**27*(2), 253–274.

[CR4] Ashby-King, D. T., & Anderson, L. B. (2022). “It gives you a better chance of getting a good job”: Memorable messages, anticipatory socialization, and first-year college students’ understandings of the purpose of college. *Communication Education,**71*(1), 2–20.

[CR5] Ashwin, P. (2020). *Transforming University Education: A Manifesto*. Bloomsbury.

[CR6] Ashwin, P. (2022). The educational purposes of higher education: Changing discussions of the societal outcomes of educating students. *Higher Education,**84*(6), 1227.

[CR7] Ashwin, P., & Trigwell, K. (2012). Evoked prior experiences in first-year university student learning. *Higher Education Research & Development,**31*(4), 449–463.

[CR8] Ashwin, P., Abbas, A., & McLean, M. (2015). Representations of a high-quality system of undergraduate education in English higher education policy documents. *Studies in Higher Education,**40*(4), 610–623.

[CR9] Ashwin, P., Abbas, A., & McLean, M. (2016). Conceptualising transformative undergraduate experiences: A phenomenographic exploration of students’ personal projects. *British Educational Research Journal,**42*(6), 962–977.

[CR10] Ashwin, P., Goldschneider, B., Agrawal, A., & Smit, R. (2023). Beyond the dichotomy of students-as-consumers and personal transformation: What students want from their degrees and their engagement with knowledge. *Studies in Higher Education*, *49*(8), 1439–1450.

[CR11] Ashworth, P. (2004). Understanding as the transformation of what is already known. *Teaching in Higher Education,**9*(2), 147–158.

[CR12] Baron, P., & McCormack, S. (2024). Employable me: Australian higher education and the employability agenda. *Journal of Higher Education Policy and Management*, *46*(3), 257–273.

[CR13] Becker, G. S. (1964). *Human capital: A theoretical and empirical analysis, with special reference to education*. University of Chicago press.

[CR14] Bernstein, B. (2000). *Pedagogy, Symbolic Control and Identity: Theory, Research and Critique* (Revised). Rowman and Littlefield Publishers.

[CR15] Biesta, G. (2022). School-as-Institution or School-as-Instrument? How to Overcome Instrumentalism without Giving Up on Democracy. *Educational Theory,**72*(3), 319–331.

[CR16] Boughey, C., & McKenna, S. (2021). *Understanding higher education: Alternative perspectives* (p. 172). Cape Town: African Minds.

[CR17] Bowden, J., & Marton, F. (1998). *The university of learning*. Kogan Page.

[CR18] Bradley, A., & Quigley, M. (2023). Governments harnessing the power of data to get ‘value for money’: A simulation study of England’s Office for Students B3 Proceed Metric. *Studies in Higher Education,**48*(8), 1289–1302.

[CR19] Brint, S. (2012). Undergraduate student orientations in the United States: Academically adrift? *Bildung und Erziehung,**65*(2), 195–207.

[CR20] Brooks, R. (2018). The construction of higher education students in English policy documents. *British Journal of Sociology of Education,**39*(6), 745–761.

[CR21] Brooks, R., & Abrahams, J. (2018). Higher education students as consumers? Evidence from England. In A. Tarabini & N. Ingram (Eds.), *Educational choices, transitions and aspirations in Europe: Systemic, institutional and subjective challenges* (pp. 185–202). Routledge.

[CR22] Brooks, R., Gupta, A., Jayadeva, S., Lainio, A., & Lažetić, P. (2022). *Constructing the higher education student: Perspectives from across Europe*. Policy Press.

[CR23] Brown, P., Lauder, H., & Cheung, S. Y. (2020). *The death of human capital? Its failed promise and how to renew it in an age of disruption*. Oxford University Press.

[CR24] Budd, R. (2017). Undergraduate orientations towards higher education in Germany and England: Problematizing the notion of ‘student as customer.’ *Higher Education,**73*(1), 23–37.

[CR25] Bunce, L., & Bennett, M. (2021). A degree of studying? Approaches to learning and academic performance among student ‘consumers.’ *Active Learning in Higher Education,**22*(3), 203–214.

[CR26] Bunce, L., Baird, A., & Jones, S. (2017). The student-as-consumer approach in higher education and its effects on academic performance. *Studies in Higher Education,**42*(11), 1958–1978.

[CR27] Case, J., Marshall, D., McKenna, S., & Mogashana, D. (2018). *Going to university: The influence of higher education on the lives of young South Africans*. Cape Town: African Minds.

[CR28] Czerniewicz, L., Mogliacci, R., Walji, S., Cliff, A., Swinnerton, B., & Morris, N. (2023). Academics teaching and learning at the nexus: Unbundling, marketisation and digitisation in higher education. *Teaching in Higher Education,**28*(6), 1295–1309.

[CR29] Dall’Alba, G., & Barnacle, R. (2005). Embodied knowing in online environments. *Educational Philosophy and Theory,**37*(5), 719–744.

[CR30] Deming, D. J. (2022). Four facts about human capital. *Journal of Economic Perspectives,**36*(3), 75–102.

[CR31] Durán Del Fierro, F. (2023). On the possibility of a public regime in higher education: Rethinking normative principles and policy frameworks. *Critical Studies in Education,**64*(2), 151–167.

[CR32] Dusi, D., & Huisman, J. (2021). It’s more complex than it seems! Employing the concept of prosumption to grasp the heterogeneity and complexity of student roles in higher education. *Higher Education,**81*(5), 935–948.

[CR33] Finney, T. G., & Finney, R. Z. (2010). Are students their universities’ customers? An exploratory study. *Education + Training,**52*, 276–291.

[CR34] Godwin, A., Potvin, G., & Hazari, Z. (2014). Do engineers beget engineers? Exploring connections between the engineering-related career choices of students and their families. *American Society for Engineering Education Annual Conference & Exposition*, 16. Indianapolis, IN: ASEE.

[CR35] Gunn, A. (2023). *Teaching Excellence? Universities in an age of student consumerism*. Sage.

[CR36] Gupta, A., Brooks, R., & Abrahams, J. (2023). Higher education students as consumers: A cross-country comparative analysis of students’ views. *Compare: A Journal of Comparative and International Education*, 1–18. 10.1080/03057925.2023.2234283

[CR37] Hooley, T. J., Bennett, D., & Knight, E. B. (2023). Rationalities that underpin employability provision in higher education across eight countries. *Higher Education,**86*(5), 1003–1023.

[CR38] Jawitz, J., & Case, J. (1998). Exploring the reasons South African students give for studying engineering. *International Journal of Engineering Education,**14*(4), 235–240.

[CR39] Lauder, H., & Mayhew, K. (2020). Higher education and the labour market: An introduction. *Oxford Review of Education,**46*(1), 1–9.

[CR40] Ljungqvist, M., & Sonesson, A. (2023). All that glitters is not gold: The depoliticization of social inequality in European education policy on ‘microcredentials’. *Journal for Critical Education Policy Studies (JCEPS)*, *21*(3). http://www.jceps.com/archives/16143

[CR41] Lonka, K., Ketonen, E., & Vermunt, J. D. (2021). University students’ epistemic profiles, conceptions of learning, and academic performance. *Higher Education,**81*(4), 775–793.

[CR42] Marginson, S. (2019). Limitations of human capital theory. *Studies in Higher Education,**44*(2), 287–301.

[CR43] Marton, F., & Booth, S. (1997). *Learning and Awareness*. Lawrence Erlbaum Associates.

[CR44] Mathebula, M. (2018). *Engineering education for sustainable development: A capabilities approach*. Routledge.

[CR45] McArthur, J. (2011). Reconsidering the social and economic purposes of higher education. *Higher Education Research & Development,**30*(6), 737–749.

[CR46] McLean, M., Abbas, A., & Ashwin, P. (2018). *How Powerful Knowledge Disrupts Inequality: Reconceptualising Quality in Undergraduate Education*. Bloomsbury.

[CR47] Mendes, A. B., & Hammett, D. (2023). The new tyranny of student participation? Student voice and the paradox of strategic-active student-citizens. *Teaching in Higher Education,**28*(1), 164–179.

[CR48] Molesworth, M., Nixon, E., & Scullion, R. (2009). Having, being and higher education: The marketisation of the university and the transformation of the student into consumer. *Teaching in Higher Education,**14*(3), 277–287.

[CR49] Molla, T., & Cuthbert, D. (2023). Crisis and policy imaginaries: Higher education reform during a pandemic. *Higher Education,**86*(1), 45–63. 10.1007/s10734-022-00899-5 PMC9360666 35968199

[CR50] Moodie, G., & Wheelahan, L. (2023). Human capital theory and its discontents. In G. Parry, M. Osborne, & P. Scott (Eds.), *Access, lifelong learning and education for all* (pp. 51–79). Palgrave Studies in Adult Education and Lifelong Learning. Palgrave Macmillan.

[CR51] Muddiman, E. (2020). Degree subject and orientations to civic responsibility: A comparative study of Business and Sociology students. *Critical Studies in Education,**61*(5), 577–593.

[CR52] Naidoo, R., & Jamieson, I. (2005). Empowering participants or corroding learning? Towards a research agenda on the impact of student consumerism in higher education. *Journal of Education Policy,**20*(3), 267–281.

[CR53] Neary, M., & Winn, J. (2009). Student as Producer. In L. Bell, H. Stevenson, & M. Neary (Eds.), *The Future of Higher Education: Policy, Pedagogy and the Student Experience* (pp. 126–138). Continuum.

[CR54] Nixon, E., Scullion, R., & Hearn, R. (2018). Her majesty the student: Marketised higher education and the narcissistic (dis) satisfactions of the student-consumer. *Studies in Higher Education,**43*(6), 927–943.

[CR55] OECD. (2017). *In-Depth Analysis of the Labour Market Relevance and Outcomes of Higher Education Systems: Analytical Framework and Country Practices Report Enhancing Higher Education System Performance*. Paris: OECD.

[CR56] Patfield, S., Gore, J., & Fray, L. (2021). On becoming a university student: Young people and the ‘illusio’of higher education. In R. Brooks R. and S. O’Shea, (Eds) *Reimagining the Higher Education Student* (pp. 10–26). London: Routledge.

[CR57] Plamper, R., Siivonen, P., & Haltia, N. (2023). Student-as-customer discourse as a challenge to equality in Finnish higher education–the case of non-fee-paying and fee-paying master’s degree students. *International Studies in Sociology of Education,**32*(1), 140–160.

[CR58] Quadlin, N., VanHeuvelen, T., & Ahearn, C. E. (2023). Higher education and high-wage gender inequality. *Social Science Research,**112*, 102873. 37061326 10.1016/j.ssresearch.2023.102873 PMC10712336

[CR59] Reynolds, A. (2022). ‘Where does my £9000 go?’ Student identities in a marketised British Higher Education Sector. *SN Social Sciences,**2*(8), 125. 35875609 10.1007/s43545-022-00432-6 PMC9297273

[CR60] Robson, J. (2023). Graduate employability and employment. In S. Marginson, B. Cantwell, D. Platonova, & B. Smolentseva (Eds.), *Assessing the Contributions of Higher Education* (pp. 177–196). Edward Elgar Publishing.

[CR61] Saunders, D. B. (2015). They do not buy it: Exploring the extent to which entering first-year students view themselves as customers. *Journal of Marketing for Higher Education,**25*(1), 5–28.

[CR62] Schäfer, G. (2024). What is higher education to contemporary students in Germany? *Higher Education Quarterly,**78*(1), 268–282.

[CR63] Scott, P. (2022). Sagas of contemporary higher education: foreground and hinterland. *Centre for Global Higher Education Working Paper Series: Working paper no. 81*. London: Centre for Global Higher Education

[CR64] Spronken-Smith, R., Buissink-Smith, N., Bond, C., & Grigg, G. (2015). Graduates’ orientations to higher education and their retrospective experiences of teaching and learning. *Teaching and Learning Inquiry,**3*(2), 55–70.

[CR65] Taylor, G. (1993). A theory of practice: Hermeneutical understanding. *Higher Education Research and Development,**12*(1), 59–72.

[CR66] Taylor Bunce, L., Rathbone, C., & King, N. (2023). Students as consumers: A barrier for student engagement. In Lowe. T (Ed) Advancing *Student Engagement in Higher Education: Reflection, Critique and Challenge.* (pp. 71 – 81). Staff and Educational Development Association Series. London: Routledge

[CR67] Tight, M. (2013). Students: Customers, clients or pawns? *Higher Education Policy,**26*(3), 291–307.

[CR68] Tomlinson, M. (2017). Student perceptions of themselves as ‘consumers’ of higher education. *British Journal of Sociology of Education,**38*(4), 450–467.

[CR69] Tomlinson, M. (2018). Conceptions of the value of higher education in a measured market. *Higher Education,**75*(4), 711–727.

[CR70] Tomlinson, M., & Watermeyer, R. (2022). When masses meet markets: credentialism and commodification in twenty-first century Higher Education. *Discourse: Studies in the Cultural Politics of Education,**43*(2), 173–187.

[CR71] Trigwell, K., & Ashwin, P. (2006). An exploratory study of situated conceptions of learning and learning environments. *Higher Education,**51*, 243–258.

[CR72] Trigwell, K., Ashwin, P., & Millan, E. S. (2013). Evoked prior learning experience and approach to learning as predictors of academic achievement. *British Journal of Educational Psychology,**83*(3), 363–378. 23822526 10.1111/j.2044-8279.2012.02066.x

[CR73] Wheelahan, L., & Moodie, G. (2022). Gig qualifications for the gig economy: Micro-credentials and the ‘hungry mile.’ *Higher Education,**83*(6), 1279–1295. 34366441 10.1007/s10734-021-00742-3 PMC8331095

[CR74] Wheelahan, L., & Moodie, G. (2024). Analysing micro-credentials in higher education: A Bernsteinian analysis. In J. Hordern, J. Muller, & D. Zongyi (Eds.), *Towards Powerful Educational Knowledge* (pp. 70–86). Routledge.

[CR75] Wheelahan, L., Moodie, G., & Doughney, J. (2022). Challenging the skills fetish. *British Journal of Sociology of Education,**43*(3), 475–494.

[CR76] Willner, T., Lipshits-Braziler, Y., & Gati, I. (2023). Construction and initial validation of the higher education orientations questionnaire. *Journal of Career Assessment*, *31*(1), 85–108.

[CR77] Wong, B., Chiu, Y. L. T., Copsey-Blake, M., & Nikolopoulou, M. (2022). A mapping of graduate attributes: What can we expect from UK university students? *Higher Education Research & Development,**41*(4), 1340–1355.

